# Prioritization of emotional faces is not driven by emotional content

**DOI:** 10.1038/s41598-022-25575-7

**Published:** 2023-01-11

**Authors:** Sjoerd M. Stuit, Chris L. E. Paffen, Stefan Van der Stigchel

**Affiliations:** Department of Experimental Psychology, Utrecht University, Utrecht, The Netherlands

**Keywords:** Consciousness, Perception, Emotion

## Abstract

Emotional faces have prioritized access to visual awareness. However, studies concerned with what expressions are prioritized most are inconsistent and the source of prioritization remains elusive. Here we tested the predictive value of spatial frequency-based image-features and emotional content, the sub-part of the image content that signals the emotional expression of the actor in the image as opposed to the image content irrelevant for the emotional expression, for prioritization for awareness. Participants reported which of two faces (displaying a combination of angry, happy, and neutral expressions), that were temporarily suppressed from awareness, was perceived first. Even though the results show that happy expressions were prioritized for awareness, this prioritization was driven by the contrast energy of the images. In fact, emotional content could not predict prioritization at all. Our findings show that the source of prioritization for awareness is *not* the information carrying the emotional content. We argue that the methods used here, or similar approaches, should become standard practice to break the chain of inconsistent findings regarding emotional superiority effects that have been part of the field for decades.

## Introduction

For any social species, the ability to convey an internal state to nearby members of the social group provides adaptational value. Displaying facial expressions is therefore thought to be important for the development of complex social structures^1,2^. As a result, expressions have become both distinct from each other (to be able to dissociate between, for example, happy versus angry) and similar and prototypical (in order to recognize the same emotion in different individuals)^3–5^. While humans demonstrate the ability to express a multitude of emotional expressions, the consensus in research into emotional expressions is that humans invariably display six discrete affects: anger, fear, disgust, happiness, surprise, and sadness^6–8^. These expressions deviate from the standard facial musculature configuration: the neutral expression. As a social cue, faces with emotional expressions attract and hold more visual attention compared to neutral expressions^9,10^. Moreover, facial expressions of emotion are thought to have prioritized access to awareness^11^.

The first evidence showing prioritized access to awareness for emotional expressions comes from binocular rivalry (BR) studies reporting that emotional expressions are more often perceived first as well as longer when in interocular conflict with neutral ones^11,12^. Furthermore, biases between different types of emotional expressions have also been shown, such as positive expressions dominating over negative expressions^13^. To avoid an influence of (conscious) attentional biases towards particular expressions, and participants anticipating what expressions are presented during a trial, more recent studies have adopted the breaking continuous flash suppression paradigm (bCFS). In this paradigm, one eye is presented with flickering mask images while a static target image is presented to the corresponding retinal locations of the other eye. The time required to first perceive the target image is interpreted as a quantification of access to awareness. The most consistent result using this paradigm is that fearful faces have prioritized access to awareness^14–20^. In addition, bCFS experiments have also replicated previous BR studies showing that positive faces are detected faster compared to negative faces^21^.

Importantly, correctly interpreting data gathered from experiments using natural images, such as facial expressions, requires accounting for all dimensions along which these images differ. Since any difference between the categories of expressions used is a valid candidate to explain observed differences in behaviour related to the expressions, knowledge about the objective image differences between expressions is crucial. This is especially relevant for research focused on visual awareness since we know that basic image-feature differences (for example spatial frequency or orientation differences) affect the temporal dynamics of interocular suppression^22–27^. For example, higher luminance contrast of an image results in shorter durations of perceptual dominance for the other image^28^, vertical orientations have longer durations of perceptual dominance compared to horizontal and oblique orientations^29^ and radial orientations have faster access to awareness compared to tangential orientations^30^. Such variation matches well with the known sensitivities of the visual system^31,32^ and previous research has suggested that such sensitivities may explain why some expressions dominate awareness more than others^19^. However, next to differences, the similarity between the two interocular images is also an important factor. For example, interocular suppression is stronger when the two images consist of similar orientations, spatial frequencies, and temporal frequencies^24,33^. Therefore, to understand why access to awareness differs between expressions, the image-properties that differ systematically between the compared expressions should be accounted for. Note that, images of emotional expressions contain visual properties that reflect the emotional content (for example the visual properties defining the shape of the mouth indicating a smile) as well as visual properties unrelated to the emotional content (for example visual properties defining the hair style or lighting conditions). Therefore, the question then arises whether the inferred meaning of the expressions has more predictive value than the basic, quantifiable visual features that make up the facial expression images. In other words, are the features that define the expressions the same as the features that drive expression-related differences in access to awareness? If not, image feature differences unrelated to emotion may have confounded previous results involving emotional faces.

Here, we ask if (1) the spatial frequency-based features that drive access to awareness the same as those that are used to label the expression of the face and if (2) the emotional content drive behaviour? We therefore test for expression-based effects on access to awareness and aim to relate such effects to the image properties of the images. Our goal is to provide more insight into the prioritization for access to awareness by predicting, using machine learning, which of two images will be perceived first. Our approach is simple: of several emotional expressions, we pinpoint (1) the image features that determine prioritization for access to awareness, (2) the image features that determine to which class of emotional expression they belong, and (3) test if the features of 1 and 2 are similar. Finally, (4) we test whether the emotional content of the expressions presented has predictive value concerning prioritization for awareness. For the current experiment, we compare access to awareness between angry, happy, and neutral faces. Not only do these expressions relate to previous research showing a bias for positive faces compared to negative faces, but they also allow for a better link to the visual search literature, where inconsistency concerning the relative speeds for finding happy versus angry faces has led to critical evaluation of the influences of differences in basic image features^34–39^.

## Results

### Initially detected image

**Figure 1:**
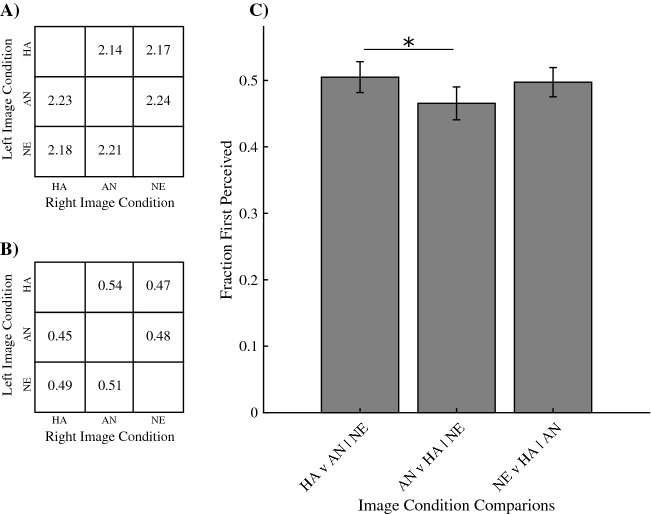
(**A**) Average median reaction time in seconds across participants per presentation condition. The y-axis shows the image class (HA: happy, AN: angry, NE: neutral) of the image presented on the left of fixation, the x-axis shows the image class of the image presented on the right of fixation. (**B**) Average fraction of the left image being perceived first across participants per presentation condition. The y-axis shows the image class of the image presented on the left of fixation with the x-axis shows the image class of the image presented on the right of fixation. (**C**) Main results showing the fractions initially detected images during bCFS (y-axis) for different presentation conditions. Specifically, from left to right on the x-axis, the fraction of happy expressions being perceived first when presented together with either angry or neutral expressions, the fraction of angry expressions being perceived first when presented together with either happy or neutral expressions, and the fraction of neutral expressions being perceived first when presented together with either happy or angry expressions. Therefore, the fraction on the y-axis is the fraction that a particular image class (on the x-axis) is detected first whenever it was presented. Results show that happy faces are detected first more frequently than angry faces (asterisk indicates significance).

To test which types of emotional facial expression are prioritized for access to awareness, participants were presented with two face images on either side of a fixation cross under conditions of continuous flash suppression. Participants were instructed to report, as soon as possible, if they detected anything other than the dynamic mask and indicate at which side of fixation this was detected. Here, (1) images of happy expressions were presented next to images of either neutral or angry expressions, (2) images of angry expressions were presented next to images of either happy or neutral expression and (3) neutral expressions were presented next to images of either happy or angry expressions. We first tested for expression-based differences in the overall fractions that an image carrying a particular expression was perceived first compared to both other expressions (Fig. 1). A Friedman ANOVA shows a main effect of Image Class (χ^2^ (2,44) = 7.1, p = 0.0287). Next, for each combination of two expressions, we tested whether one type of image was detected first more often than the other. Results showed that happy faces were detected first more frequently compared to angry faces (z = 3.13, signed rank = 172.50, p = 0.0017). No differences were found between happy and neutral (z = −0.9593, signed rank = 97, p = 0.3374) and between angry and neutral faces (z = –1.0865, signed rank = 76, p = 0.2772).

### Spatial frequency features relevant for predicting initially detected faces

**Figure 2:**
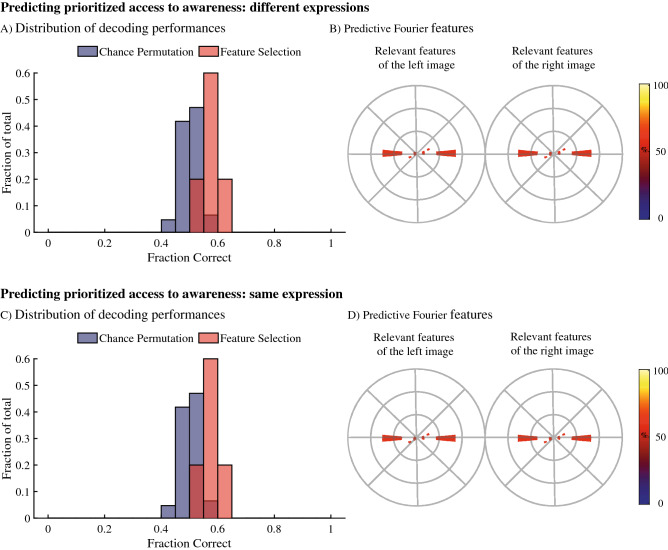
Results for decoding which image is detected first for faces with different expressions (top row) and for faces that have the same type of expression (bottom row). (**A,C**) The pool of decoding performances for the feature selection and the control models. On their y-axes is the fraction of models that show a particular performance. Chance performances from the control models are shown in purple, performances of the feature selection models are shown in red. (**B,D**) The locations of the features that were found to be significant for decoding within Fourier space for the images to the right and to the left of fixation separately. Note that in these Fourier space images, the angle between a point and the centre indicates the propagation direction of the waveform. As such, points on the horizontal axis indicate light–dark changes from left to right and therefore correspond to contrasts in vertical orientations within the images. The coloured areas in the Fourier spaces show the locations of the significant features with their colour reflecting their associated performances in percentage correct as shown in the colours on the right.

Next, we aimed to predict which image was detected first based on the spatial frequency and orientation contrasts in the two presented images (Fig. 2A,B). First, the images were converted to sets of features by segmenting the magnitude spectra of the images into 24 spatial frequency and 16 orientation ranges and summing all values per range. This results in 384 features for each of the presented images. Each feature therefore reflects the contrast energy in a particular combination of spatial frequency and orientation. Feature selection results showed that 24 out of the 768 possible features were associated with performances that exceeded the maximum chance performance based on a permutation test, corresponding to a p-value smaller than 0.001. This means that these 24 features have significant predictive value concerning which image was perceived first. These results show that the Fourier spectra of the used images contain sufficient information to predict which image was prioritized for access to awareness. The relevant information lies along the horizontal axes of the Fourier spectra and therefore corresponds to the contrast in vertical orientations in the image.

### Spatial frequency features relevant for predicting expressions in faces

**Figure 3:**
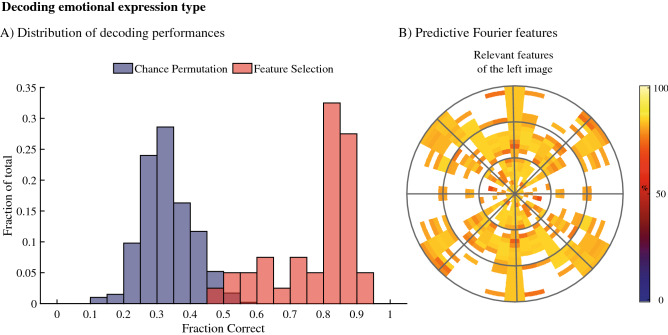
In (**A**) we show the pool of decoding performances for the feature selection and the control models. On the y-axis is the fraction of models that show a particular performance. Chance performances from the control models are shown in purple, performances of the feature selection models are shown in red. (**B**) The locations of the features that were shown significant for decoding within Fourier space. The coloured areas in the Fourier spaces show the location of the significant features with their colour reflecting their associated performances in percentage correct as shown in the colours on the right. While many orientation/spatial frequency combinations carry relevant information about the expression in the face, there is relatively little information along the horizontal axis, which reflects contrast in the vertical edges of the images.

To test whether the contrast energies—that we showed to be relevant for predicting initial access to awareness—are the same or related to the Fourier features that differentiate between emotional expressions, we used the same decoding procedure to decode the expression within a face image (Fig. 3). Note that, if predicting access to awareness is only possible due to the Fourier feature differences between the emotional faces, we expect the results in terms of relevant features to be similar to those resulting from the previous analysis. Feature selection results showed large scale differences between the expressions with 211 out of the 384 possible features being associated with performances that exceed the maximum chance performance (p-value < 0.001). These features have therefore significant predictive value over the category the images belong to. Thus, the results show that the facial expressions used in the experiment can be dissociated via their respective Fourier spectra. This raises the question as to whether the defining features of the emotional expressions (i.e., the features that drive the classification within different categories—here, happy, angry, and neutral), overlap with the defining features for predicting access to awareness (i.e., which expression is perceived first). If so, predicting access to awareness can be based on the Fourier features dissociating between the emotional expressions. If not, the features that determine which face image is perceived first is unrelated to the features that define the emotional expressions used in the images. To answer these questions, we next correlated each feature’s contributions to the performance when predicting access to awareness with the same metric but for decoding emotional expression type.

### Similarity of feature weights

To test whether a correlational approach between the weights associated with features is valid, we first correlated the feature weights in the left image with those in the right image (as a sanity check). Here, we expected a significant correlation. To do so, the weights reflecting the contribution to decoding of each feature associated with images presented on the left of fixation were correlated with those for features from images presented on the right of fixation. Features that were not tested by any of the models, and thus were assigned a weight of zero, were excluded from the data. Results show a significant positive correlation (r = 0.82, p < 0.0001), indicating that the weights of the features from images of the left eye are related to the weights of the images of the left eye. We next used the same approach to test whether predicting access to awareness is based on the same Fourier features that define the expressions in emotional faces. We therefore correlated the feature weights when decoding facial expressions to those assigned when decoding access to awareness. For the access to awareness data, the values for features from images to the left eye were averaged with the values for features from images to the right eye. Results show a non-significant correlation (r = −0.13, p = 0.096), indicating that the relevance of features for decoding access to awareness was not related to the relevance of features for decoding facial expressions. When we focus specifically on the features relevant for decoding facial expressions, and only correlate the weights of the features that are significantly involved in decoding facial expressions with the corresponding weights of the same feature but when decoding the first-perceived image during bCFS, we find a non-significant correlation with a r-value of 0.0376 with a p-value of 0.5962. These results show that access to awareness is not driven by the Fourier features that separate emotional expressions.

### Spatial frequency features relevant for predicting the initially perceived image—same expressions

Since the correlational analysis above shows no relation between the weights of the individual features when decoding emotional expression and decoding access to awareness, suggesting the stimulus properties that drove access to awareness were unrelated to emotional expression, we tested whether a difference in expressions is required to decode access to awareness (Fig. 2C,D). To do so, we repeated the original machine learning analysis with a different set of trials that consisted of two faces with the same type of expression but with different identities (either two images with happy expressions, two images with angry expressions or two images with neutral expressions presented on each side of fixation). Feature selection results show 30 out of the 768 possible features were associated with performances that exceed the maximum chance performance, corresponding to a p-value smaller than 0.001 (Fig. 2D). This means these features have significant predictive value concerning which image (e.g., identity) will be perceived first. Taken together, this indicates that, unlike decoding the expressions in the faces, and like predicting access to awareness when using two different expressions, only a small part of the Fourier content provides relevant information for decoding. Moreover, differences between expressions are not required to predict which image will be seen first.

### Predicting the initially perceived image based on expression biases

Because our results suggest emotional expressions are irrelevant for decoding behavior, we tested if the expressions indeed held no predictive value. Note that our first analysis, showing a happy superiority effect, only implies predictability of behavior, it does not test for it. Moreover, since the used non-parametric testing approach is fundamentally difference from a decoding approach, the results cannot be directly compared. To be able to do so, we predicted behavioral responses based on which type of emotional expression participants perceived first most often. Specifically, identical to our machine learning approach, we split the data in test data (10% of the data) and train data (90% of the data) ten times (folds). For each fold, we tried to predict which image in a particular combination of two expressions in the test set would be perceived first based *only* on the biases for that combination of expressions in the train set. For example, if, for the combination of happy and angry faces, the happy faces were most often perceived first in the train data, we would predict happy to be perceived first for all remaining trials with that combination in the test data. We did this comparison for each combination and found that the overall, the tenfold cross-validated performance was not significant (permutation test, *p* = 0.3615). This shows that the emotional content itself held no predictive value in our experiment. Since, the happy superiority effect likely stems from trials containing happy next to angry face images, we also tested if behavior is predictable within the subset of trials where images of happy faces are presented next to images of angry faces. Here, and since we found a happy-superiority effect, we always predicted happy to be perceived first. Results show that predictions where 54.07% correct, where p = 0.1090. Finally, we ran an analysis using all the trials where images of happy faces are presented, thus presented next to either an angry or a neutral expression, and always predicted images containing happy faces to be perceived first. There, results show that predictions where 51.24% correct, where p = 0.3640.

## Discussion

Here we show that happy expressions are prioritized over angry expressions for access to awareness, but that this effect is *unrelated* to image features that carry the information on the category of the emotional expression. The biases of the individual participants were in fact predictable, because the contrast energy of the images was sufficient to predict which image would be perceived first. The specific spatial frequency contrasts relevant for this prediction were also not the same as the specific spatial frequency contrasts relevant for decoding facial expressions. Furthermore, variation in emotional expressions were not required to predict which image would be seen first. Finally, using the participants biases to see a particular expression first had no predictive value for other trials.

The current project was mainly motivated by our interest in, and questions surrounding, emotional superiority effects. Specifically, why are emotional superiority effects consistently reported in the literature but with inconsistent results concerning what emotion is superior^36,37^? Note that our results initially added to these inconsistencies by finding a happy superiority effect. While these results are in line with research from Savage and colleagues^36^, they are inconsistent with the original emotional superiority findings of Hansen and Hansen^40^. However, on a larger scale, we do see consistent results. In a recent, comparable study, it was shown that initial eye movements between expressions can be predicted based on spatial frequency content and that there is a dissociation between prediction based on low-level image features and on emotional content^39^. Here we show a similar effect for access to awareness. In the current study, we show that vertical contrast energy is most relevant for predicting which images will be perceived first during continuous flash suppression while mainly horizontal and diagonal contrasts energies are most relevant for decoding the emotional content in the images. This difference suggests that which image is perceived first is not driven by emotional content. Ideally, a model trained to predict which image reaches awareness first is subsequently applied to images of emotional expressions to test if images containing happy faces are more likely to be predicted as first to be perceived. However, due to the differences in the number of classes and the number of features between models to predict which images will be perceived first and models that predict the emotional content in the images, the two types of models cannot be used interchangeably. Furthermore, note that images containing emotional expressions will contain both features that relate to emotional content and features that do not, complicating the interpretation of such an analysis.

Focussing on the influences of spatial frequency content, we show that decoding the emotional content within face images relies heavily on horizontal and diagonal image contrasts, while decoding which images will be perceived first relies on vertical image contrasts. Note that previous studies have shown also that horizontal, low cycles per degree, contrast energy is relevant for differentiation emotional expressions^41^, overlapping with our analysis concerning the emotional content of faces (Fig. 3) but not with our analyses concerning access to awareness (Fig. 2). The finding that vertical spatial frequency content is most relevant for predicting access to awareness (Fig. 2) is partially, but not fully, in line with a study on the depth of suppression by Yang and Blake^25^. Although caution in this comparison is required given the methodological and metric differences, we would expect to see similar results in our study and the Yang and Blake^25^ study. When inspecting the overall average Fourier magnitude spectra of all the images used in our study, a likely source for the difference between the studies is found. Specifically, in the images used in our study, the magnitudes of contrast energies are different between horizontal and vertical orientations. Moreover, the variance in these magnitudes of the horizontal and vertical contrast energies also differ. There, horizontal contrasts energies show the highest degrees of variance. Since high degrees of variance can interfere with forming predictions during machine learning, we expect the differences in variance to play a large role here. Note that other differences, for example that our metrics focus on prediction while the Yang and Blake^25^ study focuses on differences in depth of suppression distributions, and that we used checkboard masks while Yang and Blake^25^ used Mondrian masks, likely also play a role as difference between the interlocal images affect suppression^24,33^. Taken together, the patterns in our spatial frequency results are not unexpected based on previous results, further strengthening the suggestion that there is a fundamental difference between what drives an image to be seen first and the emotional content represented within those images.

Still, making a clear dissociation between the emotional content and the low-level image features that form that content remains difficult. Therefore, it is important to point out the lack of overlap in the relevant features for predicting behaviour and for decoding emotional expressions. This lack of overlap further strengthens the idea that what is driving the difference in access to awareness between two images of emotional faces in not directly related to the emotional content. Alternatively, variations in contrast sensitivity^31,32^ likely play an important role in the effects reported here and in previous studies. Since contrast sensitivity varies with spatial frequency^31^ and orientation^32^, images will vary in their stimulus strength (e.g., how sensitive humans are to them). This is especially relevant since spatial frequency and orientation content are now shown to be predictive for both initial eye movements^39^ and access to awareness between two faces, based on the spatial frequency and orientation content of the images. This explanation is in line with previous research showing that it is the effective contrast of the images, and not the emotional content, that is relevant for attracting attention^19,42^. This would also explain why face-inversion does also not reliably remove effects related to emotional expressions^37^. Moreover, given that the emotional expression type lacks predictive value, the differences in the spatial frequency and orientation contrast of the individual faces appear at least as, but likely even more, important than their categorical differences. Such differences could, for example, results from how the actor’s hair falls in different images, relative lighting angle variations, or differences in the rotation of the head. Such an interpretation fits well with previous results showing that whether a happy or an angry superiority effect is found depends on the specific models used in the experiment^37^. Note that the interpretation of emotional superiority effects, as shown to be independent of emotional content, has larger implication regarding psychological models of human behaviour. Specifically, emotion superiority effects have been taken to suggest mechanisms of early warning systems for detecting threatening information^40,43^. Given our results, and in line with Quinlan^44^ who argues that there is a lack of evidence for a threat advantage resulting from the emotional content of the face_,_ we argue against the idea of such an early warning system for threatening stimuli. Furthermore, we highlight that the stimuli used, and their image properties, are of great importance for interpreting the results.

Fortunately, dealing with the multidimensional differences between images and categories of images is more accessible than ever. With the current readily available processing power and user-friendly machine-learning software packages (for example^45,46^), visual research using natural images can, and should, go beyond comparisons between categories of images. By sorting the data based on behaviour of the participants, and not the category an image is thought to belong to, efforts should be made to find the origins of the behavioural effects. Such an approach does not focus on only testing behavioural differences but may explain why these behavioural differences occurred in the first place. In the current project, such an approach allows us to provide a different perspective on long standing issues such as processing of emotional expressions outside of awareness.

## Methods

### Participants

In this study 23 (4 males, 14 females, 5 rather not say) Dutch college students (age: *M* = 21.72 years, *SD* = 1.93) participated in return for college credits. Participants reported normal or corrected-to-normal vision, no visually triggered epilepsy or family history with epilepsy and no color blindness. All participants signed an informed consent form before taking part in the experiment. The study was approved by the local ethical committee of the faculty of social and behavioral sciences at Utrecht University. Furthermore, this research was conducted according to the principles expressed in the Declaration of Helsinki.

### Apparatus and stimuli

All stimuli were generated with MATLAB (Mathworks, Inc., USA), using Psychtoolbox^47–49^. The face stimuli used consisted of 117 faces from the Radboud Faces Database^50^ and consisted of all available photographs of adult Caucasian models with frontal-facing faces and a frontal gaze expressing the facial emotional expressions happy, angry, and neutral. All face images were converted to greyscale. The stimuli were presented on two 27-inch ASUS pb278q monitors, with a resolution of 2560 × 1440 and a frame rate of 60 Hz. A mirror stereoscope was used in front of the monitors and was angled such that a separate image was reflected onto each eye. The participant’s head was stabilized with a chin rest. The viewing distance was 45 cm. On both monitors, two pink noise square borders (8.5° × 8.5° outer edge, 5.7° × 5.7° inner edge) were presented next to each other in the center of the screen with 2.8º of space between. A fixation cross (0.3° × 0.3°) was presented at the center of the screen. During each trial, a black and white, full contrast, dynamic checkerboard mask (5.7° × 5.7°) was refreshed with a frequency rate of 10 Hz in the squares presented to one eye. The checkboard changed polarity every frame and its block size every two frames. The face stimuli (5.7° × 5.7°) that either differed in emotional expression, in model identity, or both, were presented to the other eye. The face stimuli started with 0% contrast and the contrast was gradually increased to a maximum of 20% of their maximum contrast over a period of 1 s. Note that the images were not manipulated in their contrast resulting in average maximum Michelson contrasts of 17.6% (SD: 0.3%) for happy expressions, 17.6% (SD: 0.3%) for angry expressions and 17.5% (SD: 0.3%) for neutral expressions.

### Procedure

The participants were given written and vocal instructions regarding the procedure. Participants were instructed to report as soon as possible, with the left arrow key for the left image and the right arrow key for the right image, if they detected anything other than the dynamic mask. After the instructions, participants first completed a practice run of the experiment using face images not used in the actual experiment. The experiment consisted of six conditions: happy against happy, happy against neutral, happy against angry, angry against neutral, angry against angry and neutral against neutral. Every condition had 48 trails, leading to a total of 288 trials. The stimuli were counterbalanced and randomized across trials.

### Feature extraction & labelling

To translate the face images into descriptions of their image properties, we extracted their spatial frequency information using the Protosc toolbox^47,51^ at its default settings. There, the magnitude spectra are down sampled by taking the sum of all values for 24 spatial frequency and 16 orientation ranges. This down sampling disrupts the influence of phase information, meaning that features lose all spatial specificity. With two images used per trial, this results in 768 features in total being used as the features of the subsequent machine learning analyses. The label associated with each set of features (from one trial), reflects the response given in the trial. Note that in the second machine learning analysis, where we use spatial frequency to predict the expressions in faces, the labelling reflects the category of the expression and is independent of any data generated from the participants.

### Decoding and feature selection procedures

To test whether there are spatial frequency- and orientation-based features of the images that have predictive value concerning the category to which an image belongs, we used a feature selection approach from the Protosc toolbox. This approach determines decoding performance for individual features based on the performances of a pool of cross-validated machine learning models, each allowed to select different features for decoding using Support Vector Machines. Specifically, ten models are created via a filter feature selection approach where the features with the largest differences, based on Chi-square scores between classes, are used. Next, ten models are created using a wrapper feature selection approach, which is a stepwise feature inclusion algorithm where inclusion is based on machine learning performance. Ten models are based on a random selection of features. Finally, ten models are created based on a pseudo random selection from the features that have not been used in the filter and wrapper selections created in the current fold. Note that, in each fold, the models are allowed to select a different combination of features. To calculate a distribution of chance performances, for each fold and for each method’s selection of features, twenty-five models are trained on shuffled category labels and subsequently tested on the same test data of the fold. This results in a distribution of one-thousand chance performances. The relevance of a feature is defined as the average performance of models containing the feature. Next, performances associated with the features is compared to the chance distribution. Note that not all features are tested an equal number of times. Therefore, to take into account the number of times a feature has been tested and the associated regression to the mean, a feature is determined as relevant when the average performance associated with the feature is higher than the maximum value in control distributions that also takes into account regression to the mean. All data and analyses can be found at https://osf.io/8hucp/.

## Data Availability

All data and analyses can be found at https://osf.io/8hucp/.
